# Supplementary figures and images for: 15-PGDH regulates hematopoietic and gastrointestinal fitness during aging
Source: PLoS One. 2022 May 19;17(5):e0268787. doi: 10.1371/journal.pone.0268787 (PMC9119474; doi:10.1371/journal.pone.0268787)

**S1 Graphical abstract**

**
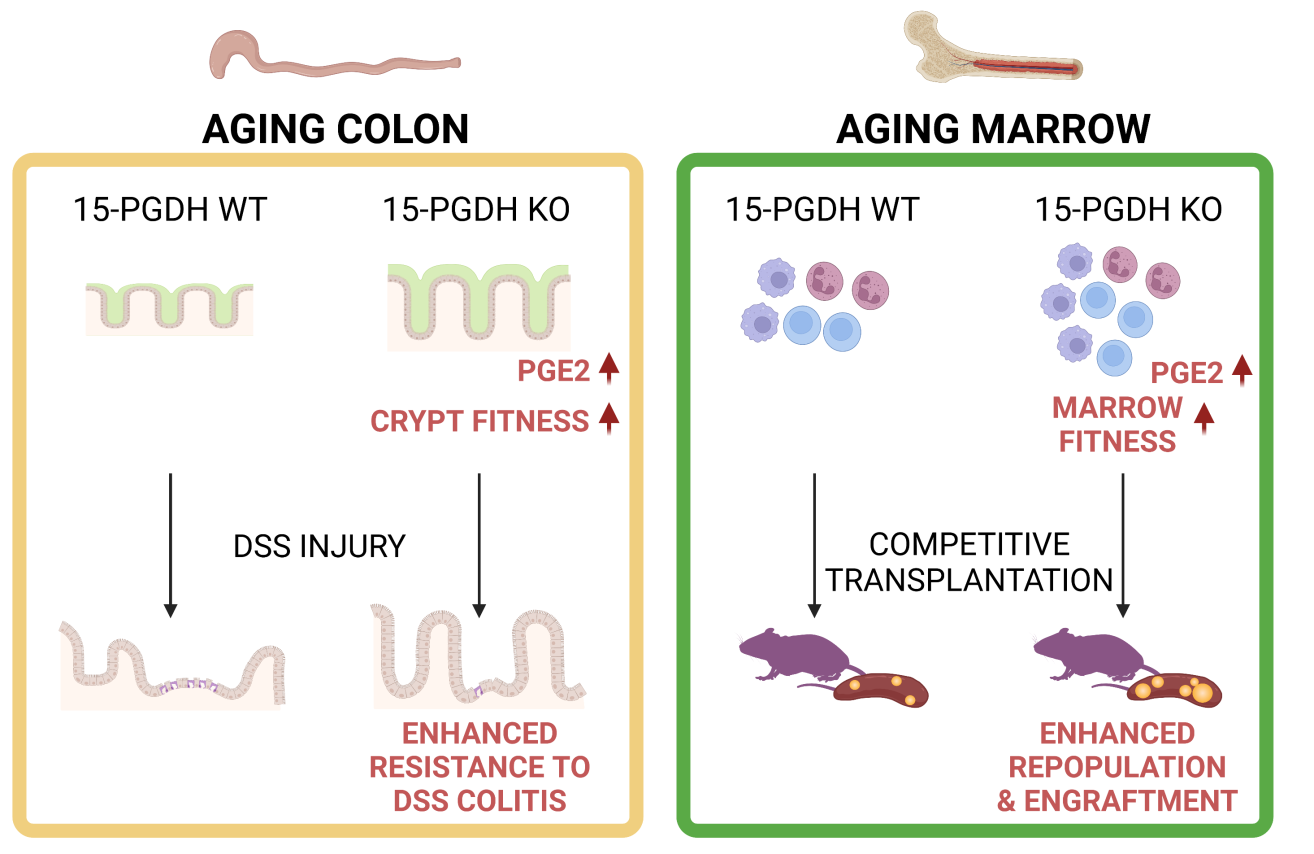
**

**(Created with BioRender.com)**

Supplement: S1 Graphical abstract — (DOCX) [file pone.0268787.s001.docx]

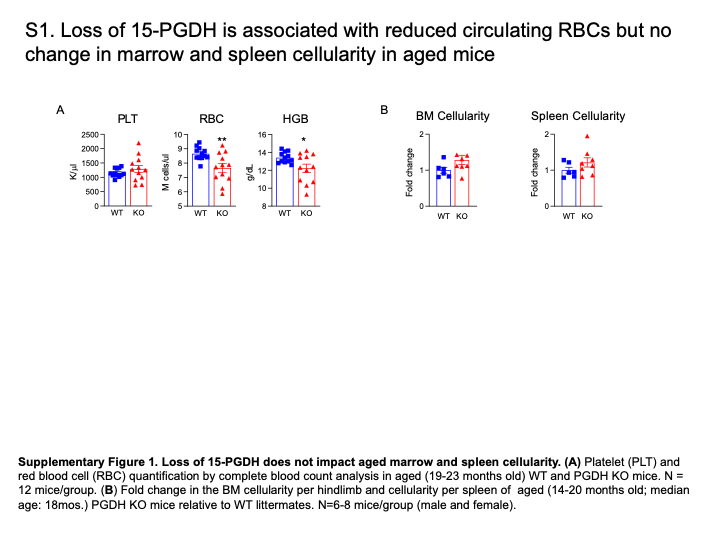

Supplement: S1 Fig — (A) Platelet (PLT), red blood cell (RBC), and hemoglobin (HGB) quantification by complete blood count analysis in aged (19–23 months old) WT and PGDH KO mice. N = 12 mice/group. (B) Fold change in the BM cellularity per hindlimb and cellularity per spleen of aged (14–20 months old; median age: 18mos.) PGDH KO mice relative to WT littermates. N = 6–8 mice/group. (male and female). (TIFF) [file pone.0268787.s002.tiff]

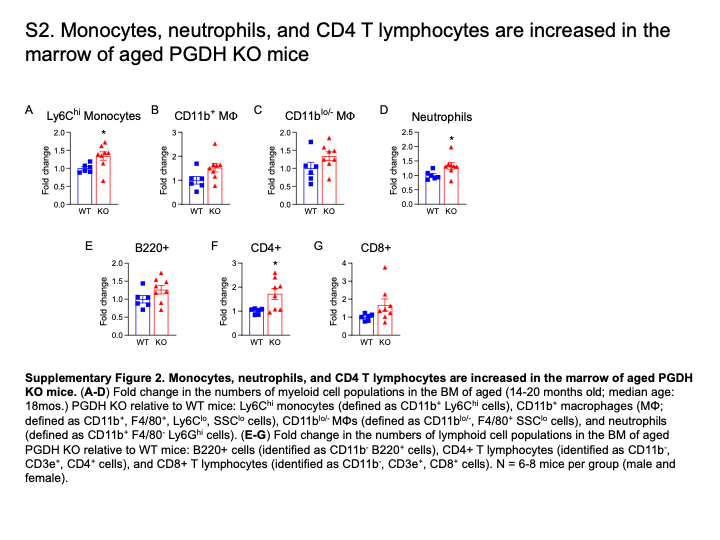

Supplement: S2 Fig — (A-D) Fold change in the numbers of myeloid cell populations in the BM of aged (14–20 months old; median age: 18mos.) PGDH KO relative to WT mice: Ly6Chi monocytes (defined as CD11b+ Ly6Chi cells), CD11b+ macrophages (MΦ; defined as CD11b+, F4/80+, Ly6Clo, SSClo cells), CD11blo/- MΦs (defined as CD11blo/-, F4/80+ SSClo cells), and neutrophils (defined as CD11b+ F4/80- Ly6Ghi cells). (E-G) Fold change in the numbers of lymphoid cell populations in the BM of aged PGDH KO relative to WT mice: B220+ cells (identified as CD11b- B220+ cells), CD4+ T lymphocytes (identified as CD11b-, CD3e+, CD4+ cells), and CD8+ T lymphocytes (identified as CD11b-, CD3e+, CD8+ cells). N = 6–8 mice per group. (male and female). (TIFF) [file pone.0268787.s003.tiff]

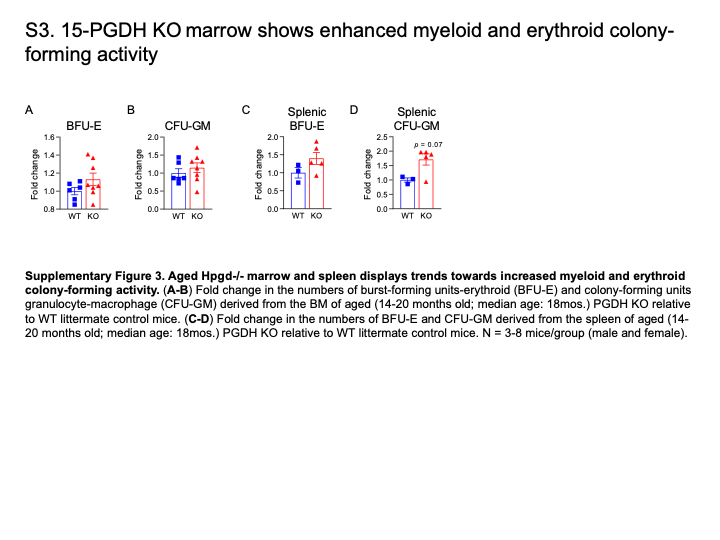

Supplement: S3 Fig — (A-B) Fold change in the numbers of burst-forming units-erythroid (BFU-E) and colony-forming units granulocyte-macrophage (CFU-GM) derived from the BM of aged (14–20 months old; median age: 18mos.) PGDH KO relative to WT littermate control mice. (C-D) Fold change in the numbers of BFU-E and CFU-GM derived from the spleen of aged (14–20 months old; median age: 18mos.) PGDH KO relative to WT littermate control mice. N = 3–8 mice/group. (male and female). (TIFF) [file pone.0268787.s004.tiff]

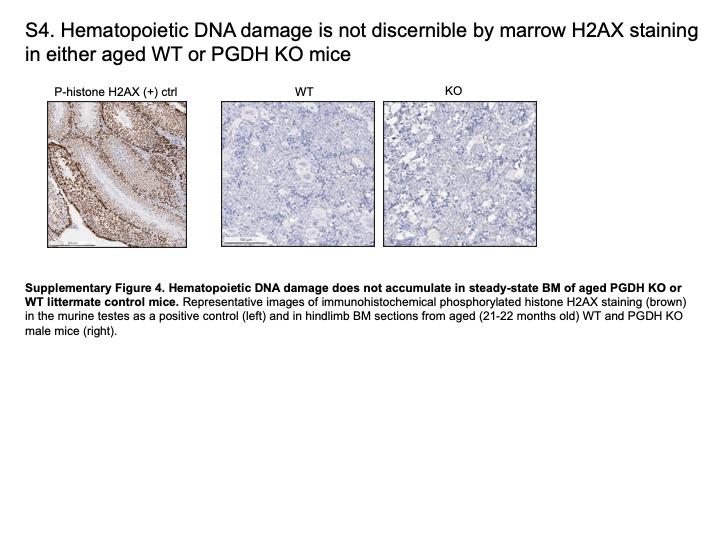

Supplement: S4 Fig — Representative images of immunohistochemical phosphorylated histone H2AX staining (brown) in the murine testes as a positive control (left) and in hindlimb BM sections from aged (21–22 months old) WT and PGDH KO male mice (right). (TIFF) [file pone.0268787.s005.tiff]

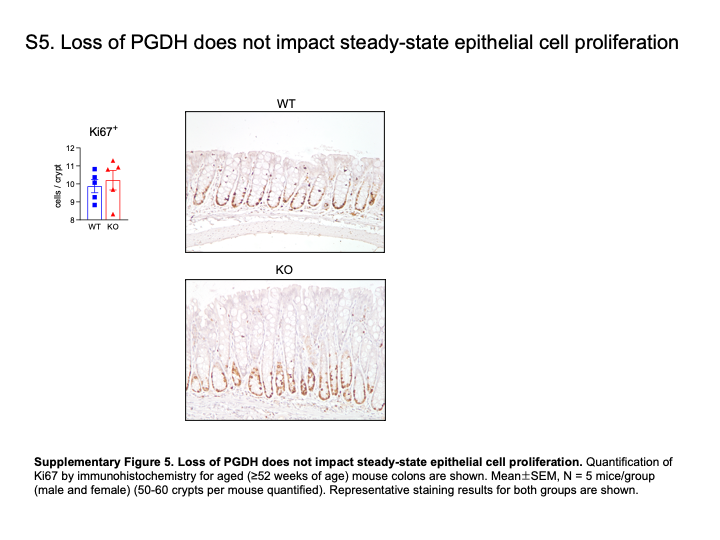

Supplement: S5 Fig — Quantification of Ki67 by immunohistochemistry for aged (≥12 months of age) mouse colons are shown. Mean±SEM, N = 5 mice/group (male and female). (50–60 crypts per mouse quantified). Representative staining results for both groups are shown. (TIFF) [file pone.0268787.s006.tiff]

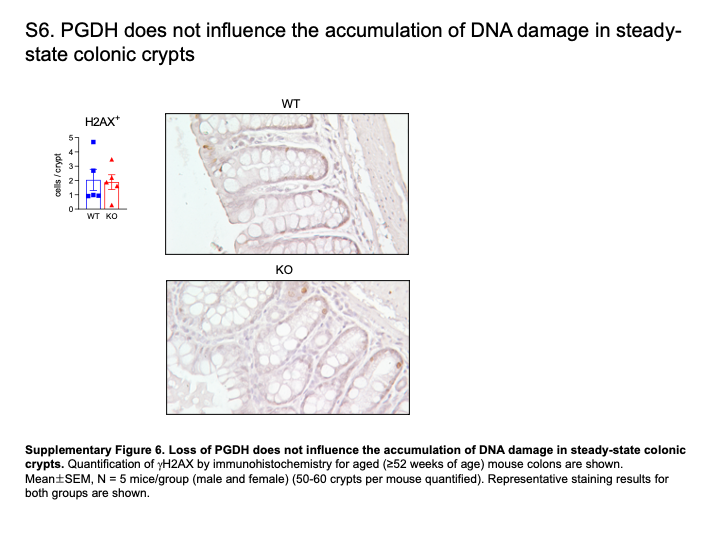

Supplement: S6 Fig — Quantification of γH2AX by immunohistochemistry for aged (≥12 months of age) mouse colons are shown. Mean±SEM, N = 5 mice/group (male and female). (50–60 crypts per mouse quantified). Representative staining results for both groups are shown. (TIFF) [file pone.0268787.s007.tiff]

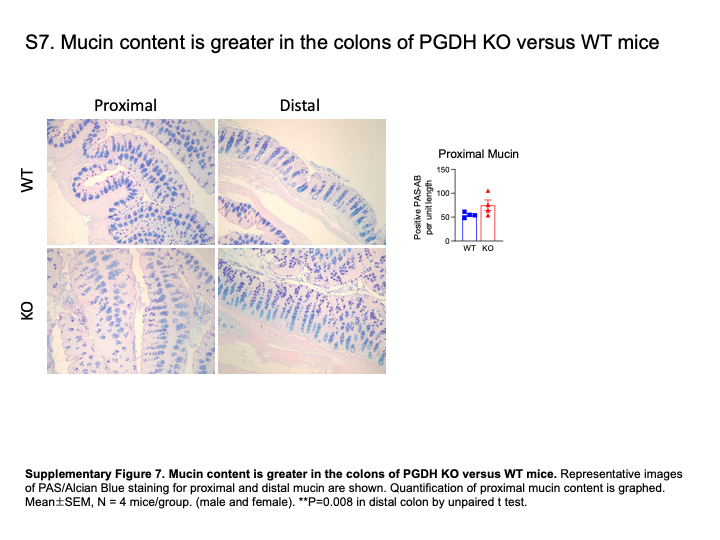

Supplement: S7 Fig — Representative images of PAS/Alcian Blue staining for proximal and distal mucin are shown. Quantification of proximal mucin content is graphed. Mean±SEM, N = 4 mice/group. (male and female).**P = 0.008 in distal colon by unpaired t test. (TIFF) [file pone.0268787.s008.tiff]

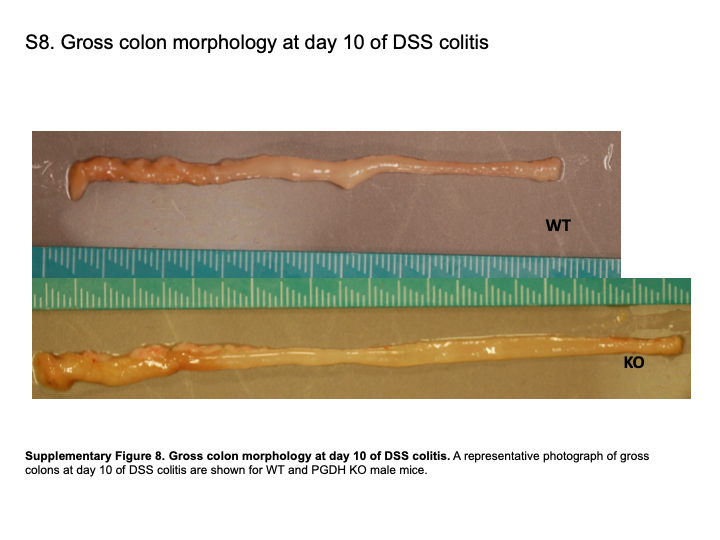

Supplement: S8 Fig — A representative photograph of gross colons at day 10 of DSS colitis are shown for WT and PGDH KO male mice. (TIFF) [file pone.0268787.s009.tiff]

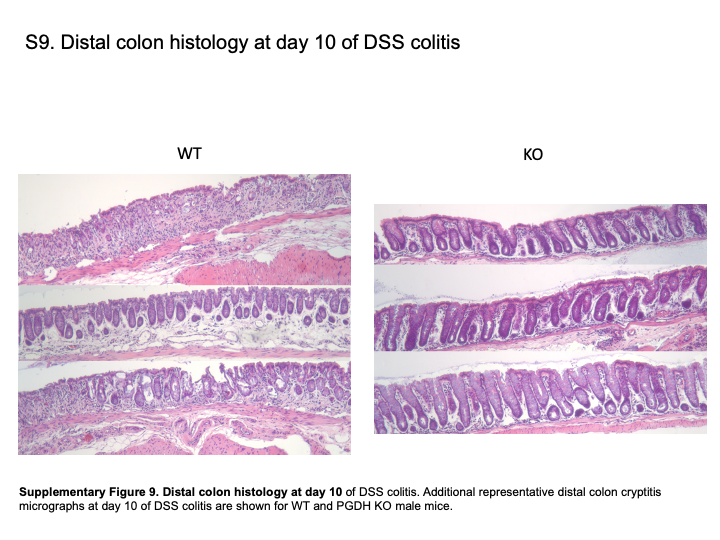

Supplement: S9 Fig — Additional representative distal colon cryptitis micrographs at day 10 of DSS colitis are shown for WT and PGDH KO male mice. (TIFF) [file pone.0268787.s010.tiff]

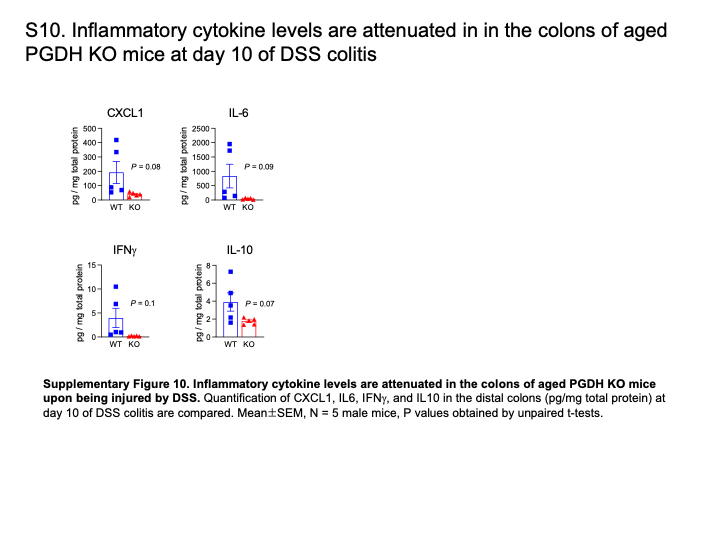

Supplement: S10 Fig — Quantification of CXCL1, IL6, IFNγ, and IL10 in the distal colons (pg/mg total protein) at day 10 of DSS colitis are compared. Mean±SEM, N = 5 male mice, P values obtained by unpaired t-tests. (TIFF) [file pone.0268787.s011.tiff]

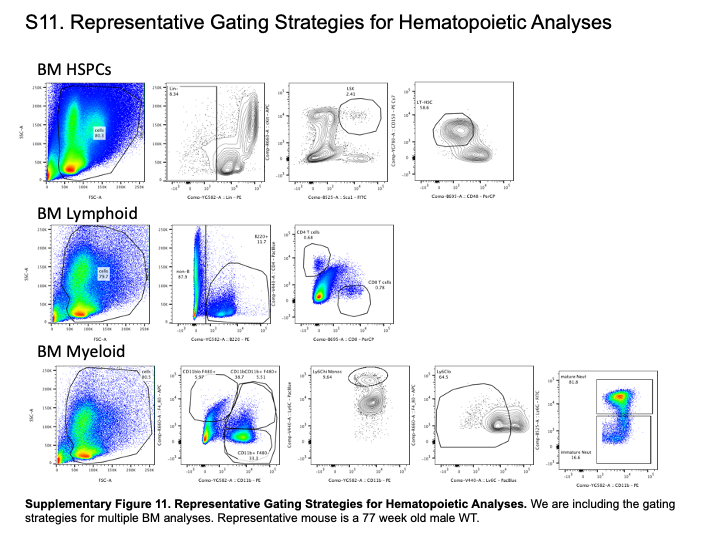

Supplement: S11 Fig — We are including the gating strategies for multiple BM analyses. Representative mouse is a 77-week-old male WT. (TIFF) [file pone.0268787.s012.tiff]

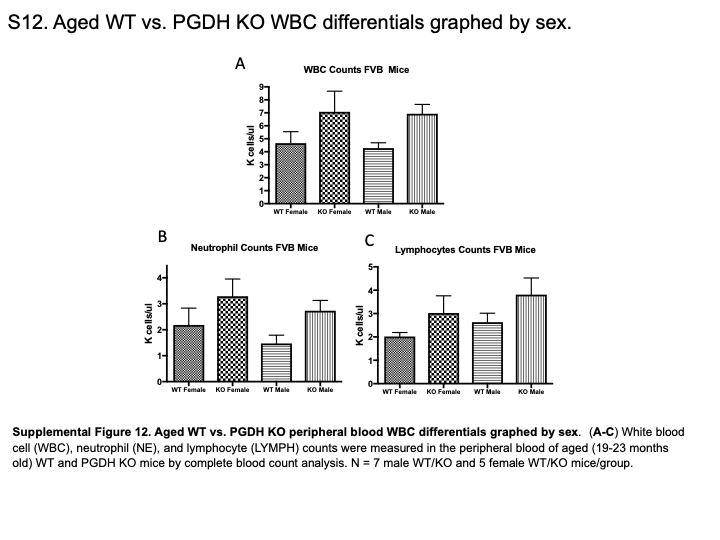

Supplement: S12 Fig — (A-C) White blood cell (WBC), neutrophil (NE), and lymphocyte (LYMPH) counts were measured in the peripheral blood of aged (19–23 months old) WT and PGDH KO mice by complete blood count analysis. N = 7 male WT/KO and 5 female WT/KO mice/group. (TIFF) [file pone.0268787.s013.tiff]
